# Ms gene and Mr gene: Microbial‐mediated spatiotemporal communication between plants

**DOI:** 10.1002/imt2.210

**Published:** 2024-06-07

**Authors:** Ming‐Hao Lv, Wen‐Chong Shi, Ming‐Cong Li, Bo Zhou, Yong‐Xin Liu, Zheng Gao

**Affiliations:** ^1^ College of Life Sciences Shandong Agricultural University Tai'an Shandong China; ^2^ Shenzhen Branch, Guangdong Laboratory of Lingnan Modern Agriculture Genome Analysis Laboratory of the Ministry of Agriculture and Rural Affairs, Agricultural Genomics Institute at Shenzhen, Chinese Academy of Agricultural Sciences Shenzhen Guangdong China

## Abstract

Within dynamic agroecosystems, microbes can act as key intermediaries, facilitating spatiotemporal communication among plants. Future research could categorize key plant genes involved in plant–microbe interactions into microbiome‐shaping genes (Ms genes) and microbiome‐responsive genes (Mr genes), potentially leading to the construction of spatiotemporal molecular networks with microbes as intermediaries.

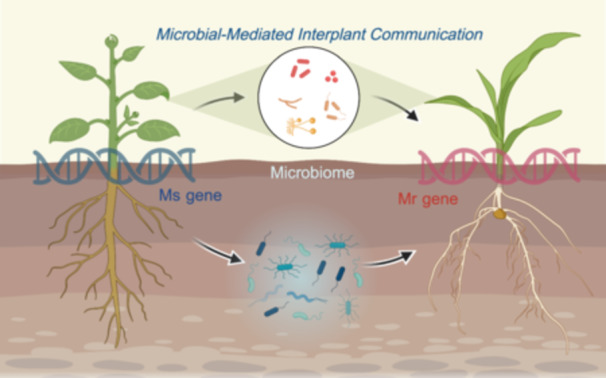

Over the course of 450 million years, terrestrial plants and microbes have coevolved, which is now reflected in the complexity of contemporary plant–microbe interactions [1]. Plants provide a plethora of ecological niches for a variety of microbial entities. In turn, these plant‐regulated microbes can form complex symbiotic relationships, significantly impacting plant health. Recently, the term “M genes” has been employed to describe plant genes crucial in shaping microbial communities [2]. This provides new insights into gene–phenotype relationships, indicating that some genes may influence associated microbial communities and, thus, indirectly affect plant phenotypic variability via microbe interactions. Some genes work in a “top‐down” fashion to shape communities, while others are easily influenced by microbes, creating a “bottom‐up” causation of plant phenotypic variations [3]. To further elucidate the microbial mediation in plant adaptation to the environment, this study proposes a categorization of host genes closely associated with microbes into microbiome‐shaping genes (Ms genes) and microbiome‐responsive genes (Mr genes).

## UNDERSTAND Ms GENES: CHALLENGES AND FUTURE PERSPECTIVES

The influence of Ms genes on plant‐associated microbes can manifest through several facets, such as the modulation of specific small peptides and secondary metabolites and the trans‐kingdom transfer of miRNAs. For example, *OsPAL02* has been identified as playing a pivotal role in maintaining the equilibrium of the phyllosphere microbiome through its biosynthetic function for 4‐hydroxycinnamic acid (4‐HCA) [2, 4]. Additionally, plant volatile organic compounds (VOCs) can regulate the microbial communities on plant surfaces through their antimicrobial properties and by serving as carbon sources for certain microbes. In turn, the shaped phyllosphere microbial communities also possess the potential to influence plant physiology [5]. In natural ecosystems, these mechanisms may not operate in isolation but rather through a complex and multifaceted regulatory network to shape specific microbial communities.

Plant metabolomic changes are highly dynamic and complex, influenced profoundly by plant genotype, developmental stage, and environmental factors. This implies that Ms genes involved in secondary metabolite synthesis or release pathways might be particularly sensitive to environmentally related changes, enabling plants to shape beneficial microbial communities for better environmental adaptability. For example, coumarins secreted by plant roots in iron‐limited soils can stimulate rhizosphere microbial growth and activity; these microbes can assist in iron uptake by plants, enhancing their iron nutrition [6]. It is crucial to note that not all secondary metabolite releases regulating microbes are beneficial to plants; such regulatory effects of plant secondary metabolites on microbes may stem from incidental responses of certain genes to the environment, not functionally enhancing plant environmental adaptability, that is, nonaltruistic microbial shaping patterns. We favor an interpretation and application of the Ms gene concept that does not overly generalize its purview. We contend that the term Ms gene should be directed toward addressing a relatively complete biological process, whereby the microbiome molded by the Ms gene bears significant biological implications to plant or soil ecology.

Although the preceding description elucidates key pathways through which plant Ms genes shape the associated microbiome, there remain intriguing mechanisms by which Ms genes regulate microbes that warrant further attention, such as miRNAs [7] and circadian rhythms [8]. Studies have demonstrated that plant miRNAs can be transferred to fungal pathogens via extracellular vesicles, silencing the pathogen's virulence genes [9]. Interestingly, plant circadian rhythms have also been found to reshape rhizosphere microbial communities. Research has shown that approximately 13% of the microbial community exhibits circadian rhythmicity [8].

**Figure 1:**
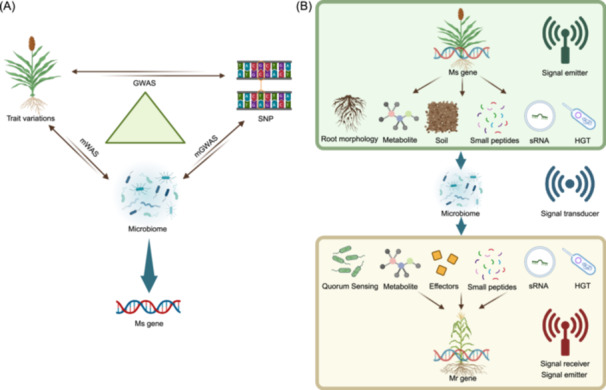
Microbial‐mediated plant communication. (A) Through the combined analysis of genome‐wide association studies (GWAS), microbiome genome‐wide association studies (mGWAS), and microbiome‐wide association studies (mWAS), researchers identified potential Ms genes by investigating the relationships among host genetics, microbiomes, and host phenotypes. (B) Pattern diagram of microbial‐mediated plant communication across time and space. Initially, the plant's Ms genes function as “signal generators,” shaping the microbial community through various mechanisms, including influencing plant root architecture, releasing metabolites, altering soil structure, cross‐kingdom regulation by miRNA, and horizontal gene transfer. Subsequently, within intercropping systems, microbes act as “signal storage” or “signal transducer,” capturing the “language” emitted by plants, processing it, and then relaying it back to the plants. In crop rotation systems, microbes have the capacity to temporarily “store” information, serving as a soil legacy, which modulates the growth and development of subsequent plant generations. The graphic was created with BioRender.com.

Genome‐wide association studies (GWASs) have unveiled numerous linkages between plant phenotypes and single‐nucleotide polymorphisms (SNPs). Recently proposed concepts like microbiome‐wide association studies (mWAS) and microbiome genome‐wide association studies (mGWAS) aim to address the complexities of plant phenotypic variation within natural ecosystems that cannot be wholly explained by GWAS alone [10]. Combining GWAS, mWAS, and mGWAS is essential for researching host genetics, microbiomes, and traits, which will significantly aid in the discovery of Ms genes and guide the subsequent identification of these genes through overexpression, mutant strains, and sterile soil controls (Figure 1A). In addition, it is noteworthy that the assembly processes of plant‐associated microbiomes, particularly phyllosphere microbiota, are inevitably subjected to profound disturbances by various environmental factors, which can significantly interfere with precision shaping from Ms genes to the microbiome. This implies that the functional design of microbiomes necessitates the support of a more comprehensive framework, integrating approaches such as holo‐omics, genetic manipulation, and culture‐dependent characterization [11].

It is thought‐provoking to consider that the plant's capacity to utilize microbial resources is deeply influenced by native dominant microorganisms in the soil, posing a challenge for the practical application of Ms genes due to environmental fluctuations, even if some microbial communities are vertically transmitted via plant seeds. A viable solution could involve employing microbial inoculants to regulate soil microbial communities, enabling optimal function of Ms genes and exploiting their “home‐field advantage,” thereby achieving and optimizing plant genetic engineering tailored to the microbiome. This means that the practical applications of Ms genes in the future must consider the environmental characteristics of the specific microbial taxa shaped by Ms genes. On the other hand, the discovery of Ms genes should complement the study of plant core microbiomes. Current research predominantly defines core microbiomes based on higher occurrence frequency and relative abundance within samples, a widely accepted method. Undoubtedly, exploring and engineering plant Ms genes targeting the core microbiome may have more universal application potential. Ms genes associated with the core microbiome could alleviate the pressures of regional application constraints to some extent. Nonetheless, it is crucial to recognize that rare microbes, once overlooked, are increasingly considered vital yet fragile components of the microbial community. These low‐abundance microbes may play crucial roles in maintaining beneficial host–plant relationships [12]. In essence, these studies remind us that the rare and low‐abundant “noncore” microbes shaped by Ms genes may also play a significant role in host health. Another noteworthy point is the functional expression regulation of plant‐associated microbes by Ms genes. Instead of just utilizing abundance data, exploring omics techniques such as metatranscriptomics is strongly suggested for future Ms gene studies, with a greater focus on microbial function than on their abundance.

## UNDERSTAND Mr GENES: CHALLENGES AND FUTURE PERSPECTIVES

Within ecosystems, certain genes in plants exhibit heightened sensitivity to the activity of microbes or to specific molecular signals released by them, granting microbes the capacity to regulate plant adaptation to environmental stressors [13]. These genes are termed Mr genes. Arbuscular mycorrhizal fungi (AMF) could enhance plant drought resistance by modulating the expression of the 9‐cis‐epoxycarotenoid dioxygenase (NCED) genes [3]. A specific milRNA in the stripe rust fungus has been identified to be exported to wheat following infection, whereby it suppresses the wheat pathogenesis‐related 2 gene [14]. Moreover, in the aerial parts of plants, key microbial communities associated with disease‐resistant rice panicles have been identified to suppress pathogen infection by enhancing the content of the branched‐chain amino acid (BCAA) in the host panicles [15]. Overall, Mr genes assist the host in adapting to environmental changes by receiving specific signals from microbes. Notably, under biotic stress, Mr genes represent a unique class, which, upon sensing pathogens, rapidly transmit alarm signals downstream to assist the plant in recruiting beneficial microbial communities to defend aganist pathogenic invasions, forming part of a specialized plant defense signaling pathway. In this context, we postulate the potential existence of feedback loops in the Ms gene‐ microbiome‐Mr gene regulation, with some genes possibly acting as both regulators and effectors. This implies that Mr genes under biotic stress could act as Ms genes to regulate the next Ms gene‐microbe‐Mr gene‐induced plant resistance process. A classic example is the enrichment of oligotrophic *Pseudomonas* in the wheat rhizosphere following *Fusarium* infection, which in turn promotes large‐scale expression of genes involved in salicylic and jasmonic acid signaling pathways, enhancing the host's disease immunity [16]. Like Ms genes, we advocate for a standardized use of the concept of Mr genes in future research, as a multitude of genes may experience strong, incidental influences triggered by the microbiome, while only a subset (Mr genes) sensitively perceives the “language” of microbes to modulate plant environmental adaptability.

In future work, Mr genes can be incorporated into microbiome‐centered genetic engineering. Specifically, some plant varieties may lack Mr genes, which are targets of microbial regulation, leading to poorer environmental adaptability in certain varieties. This means that the beneficial regulatory functions of Ms genes on plant varieties are fundamentally inseparable from the presence of corresponding Mr genes. Additionally, engineering breeding targeting Mr genes may offer unique advantages since the enactment of Mr gene functions is passive and can be “bottom‐up” regulated by environmental microbial communities. In other words, even without Ms gene regulation, the potential function of Mr genes can be sufficiently exploited by improving the native soil microbial resources. Hence, this reminds us that improving the microbial environment around the host, such as through seed treatment with microbial inoculants or applying inoculants to the soil, could shape a special microbial resource environment and induce the function of Mr genes.

## LINK Ms AND Mr GENES: MICROBIAL‐MEDIATED PLANT COMMUNICATION

Agricultural intensification has ushered in discernible sustainability crises within agroecosystems, emphasizing the need to reevaluate the significance of diversified planting strategies—such as intercropping or crop rotation—under temporal and spatial considerations. In agricultural systems, different cultivation methods noticeably influence plant traits. For example, intercropping tomatoes with scallions can substantially suppress tomato wilt by modulating soil *Bacillus* communities [17].

The introduction of Ms gene and Mr gene concepts offers profound insights into plant‐to‐plant communication within varying planting schemes, such as intercropping, monoculture, and crop rotation. Microorganisms act as connectors in this plant interaction network across different temporal/spatial dimensions (Figure 1B). Initially, the expression of Ms genes is contingent upon the secretion of signaling substances by plant roots, including flavonoids or triterpenoids, or through other mechanisms such as altering root architecture [3] and horizontal gene transfer [18], thus establishing unique plant‐associated microbial consortia. Following this, certain microbes intercept pivotal signals from plants. These influenced microbes might, in turn, regulate the plants themselves and other spatially proximate plants via released VOCs or quorum‐sensing signaling molecules [3].

We consider that in ecosystems, this mode of communication, extending from plants to the environment and vice versa, can generate extensive cascading effects. In other words, each plant's Ms genes within the ecosystem functions as a signal emitter, its message translated through environmental microbes and affecting adjacent plants. Plants receive this signal via the Mr genes process and then perpetuate further signal transmission. Notably, in crop rotation systems, adjusted microbes persist as “soil legacies,” influencing the regulatory processes for the growth of subsequent plant generations. Here, the ecosystem's microbes bridge the communication between plants across diachronic and spatial dimensions, where the Ms gene effect on the Mr gene may demonstrate a delayed response due to spatial and temporal separations. Significantly, the regulatory network model of “Ms genes‐microbes‐Mr genes” may provide a crucial reference for the design of “precision agriculture” in the future. The exploration, connection, and identification of Ms genes, key functional microbial taxa, and Mr genes can facilitate personalized crop variety selection in the precision design of diversified cropping systems (such as intercropping or crop rotation). Subsequently, by focusing on potential key microbial groups that act as pivotal hubs within the “Ms genes‐microbes‐Mr genes” regulatory network model, personalized microbial agents can be designed. This strategic integration of carefully selected crop varieties alongside these personalized microbial agents will set the stage for realizing elevated agricultural efficiency in the future. This microbial‐mediated interspatial communication pattern among plants highlights the potential for “altruistic” breeding strategies that focus on enhancing the overall agricultural ecosystem rather than optimizing individual varieties, which may help address current breeding challenges. In addition, rapid advances in high‐throughput sequencing technologies such as metagenomics and transcriptomics now offer the potential to establish directed regulatory networks between inter‐species Ms gene‐microbe‐Mr gene interactions under intercropping or crop rotation systems. Importantly, constructing such regulatory networks is anticipated to be complex and challenging. Future research will likely be impacted by environmental factors, including soil physicochemical properties, temperature, and plant residues, which can significantly perturb the robustness of networks involving Ms gene‐microbe‐Mr gene interactions.

## AUTHOR CONTRIBUTIONS

Ming‐Hao Lv and Wen‐Chong Shi drafted the paper. Zheng Gao, Yong‐Xin Liu, Ming‐Cong Li, and Bo Zhou provided guidance throughout the preparation of this manuscript. All authors have read the final manuscript and approved it for publication.

## CONFLICT OF INTEREST STATEMENT

The authors declare no conflict of interest.

## ETHICS STATEMENT

No animals or humans were involved in this study.

## Data Availability

This manuscript does not generate any code or data. Supplementary materials (graphical abstract, slides, videos, Chinese translated version, and updated materials) may be found in the online DOI or iMeta Science http://www.imeta.science/.
